# Sequence analysis of Epstein-Barr virus (EBV) early genes BARF1 and BHRF1 in NK/T cell lymphoma from Northern China

**DOI:** 10.1186/s12985-015-0368-3

**Published:** 2015-09-04

**Authors:** Lingling Sun, Kui Che, Zhenzhen Zhao, Song Liu, Xiaoming Xing, Bing Luo

**Affiliations:** Department of Pathology, Affiliated Hospital of Qingdao University, 16 Jiangsu Road, Qingdao, 266003 China; Department of Medical Microbiology, Qingdao University Medical College, 38 Dengzhou Road, Qingdao, 266021 China; Department of Clinical Laboratory, Affiliated Hospital of Qingdao University, 1677 Wutaishan Road, Qingdao, 266555 China

## Abstract

**Background:**

NK/T cell lymphoma is an aggressive lymphoma almost always associated with EBV. BamHI-A rightward open reading frame 1 (BARF1) and BamHI-H rightward open reading frame 1 (BHRF1) are two EBV early genes, which may be involved in the oncogenicity of EBV. It has been found that V29A strains, a BARF1 mutant subtype, showed higher prevalence in NPC, which may suggest the association between this variation and nasopharyngeal carcinoma (NPC). To characterize the sequence variation patterns of the Epstein-Barr virus (EBV) early genes and to elucidate their association with NK/T cell lymphoma, we analyzed the sequences of BARF1 and BHRF1 in EBV-positive NK/T cell lymphoma samples from Northern China.

**Methods:**

In situ hybridization (ISH) performed for EBV-encoded small RNA1 (EBER1) with specific digoxigenin-labeled probes was used to select the EBV positive lymphoma samples. Nested-polymerase chain reaction (nested-PCR) and DNA sequence analysis technique were used to obtain the sequences of BARF1 and BHRF1. The polymorphisms of these two genes were classified according to the signature changes and compared with the known corresponding EBV gene variation data.

**Results:**

Two major subtypes of BARF1 gene, designated as B95-8 and V29A subtype, were identified. B95-8 subtype was the dominant subtype. The V29A subtype had one consistent amino acid change at amino acid residue 29 (V → A). Compared with B95-8, AA change at 88 (L → V) of BHRF1 was found in the majority of the isolates, and AA79 (V → L) mutation in a few isolates. Functional domains of BARF1 and BHRF1 were highly conserved. The distributions of BARF1 and BHRF1 subtypes had no significant differences among different EBV-associated malignancies and healthy donors.

**Conclusion:**

The sequences of BARF1 and BHRF1 are highly conserved which may contribute to maintain the biological function of these two genes. There is no evidence that particular EBV substrains of BARF1 or BHRF1 is region-restricted or disease-specific.

## Background

Epstein-Barr virus (EBV) is a member of gamma herpes virus family and persistently infects B lymphocytes in more than 90 % population of adults [1]. EBV is related to the tumorigenesis of various malignancies, which include some epithelial cell malignancies, such as nasopharyngeal carcinoma (NPC) and EBV-associated gastric carcinoma (EBVaGC), and a variety of lymphocytic cell malignancies, including Burkitt lymphoma (BL), Hodgkin lymphoma (HL), and post-transplant and AIDS associated lymphoproliferative disorders [2, 3].

Although being a B-lymphotropic virus, EBV can also infect NK/T cells [4] and is highly associated with natural killer (NK)/T cell lymphoma [5]. NK/T cell lymphoma derives from natural killer (NK) cells or activated γδ or αβ cytotoxic T cells (CTLs) and expresses granzyme B, TIA-1 and perforin [6]. Unlike B-cell lymphomas, EBV-associated NK/T cell lymphoma seems to be site-restricted. EBV is found in nearly 100 % nasal NK/T cell lymphoma but rarely in primary cutaneous NK/T cell lymphoma [6].

Despite the ubiquity of the EBV infection, the frequencies of EBV-associated malignancies differ in different geographic regions, which may suggest that particularly tumorigenic EBV strains might exist. Studies have been carried out to determine variations in EBV genome and explore their relationship with NPC, EBVaGC or other EBV-associated disorders. Just like other EBV-associated malignancies, the frequency of NK/T lymphoma varies in different geographic regions [7]. But only a few studies have investigated EBV gene variations in NK/T cell lymphoma.

BamHI-A rightward open reading frame 1 (BARF1) and BamHI-H rightward open reading frame 1 (BHRF1) are two EBV early genes critical to replication of the virus, encoding proteins homologous to important human proteins c-fms and Bcl-2 respectively. Recently, the role of BARF1 and BHRF1 in the development of EBV associated tumors has drawn great interest. Both transcripts of them were detected in NK/T cell lymphoma [8, 9].

BARF1 gene is a multifunctional gene. It could induce malignant transformation in rodent fibroblasts [10] and enhance the tumorigenicity of EBV-negative Louckes and Akata cells [11, 12]. The first 54 amino acids at the N-terminus may be responsible for the malignant transformation of BARF1 [10]. In addition, this region could also upregulate the cellular anti-apoptotic protein Bcl-2. The secreted hexameric BARF1-encoded protein has immune modulation properties. It is a homologue of c-fms, the human colony stimulating factor 1 (hCSF-1) receptor and has the ability to bind CSF-1 [13] therefore inhibiting interferon-alpha secretion from mononuclear cells. The immune modulation ability of BARF1 allowing EBV-infected tumor cells to escape elimination of host. Despite its immune-modulating properties, BARF1 protein may trigger an immune response as a target for antibody-dependent cytotoxicity [14]. Several HLA-A*0201-restricted cytotoxic T lymphocyte (CTL) epitopes of BARF1 have been identified [15].

Though it was widely believed that BARF1 expressed frequently in latently infected carcinomas and rarely in lymphomas [16, 17], some studies detected the expression of BARF1 in latently infected B cells [18] and B lymphoma in Malawi [19]. Zhang et al. [8] detected BARF1 expression in nasal NK/T lymphoma and postulated that BARF1 expression might be associated with the pathogenesis in NK/T cell lymphoproliferative disorders [8].

BHRF1 is structurally and functionally homologous to the pro-survival protein Bcl-2. It could protect T and B lymphocytes from apoptosis induced by growth factor withdrawal, chemotherapeutic drug or granzyme B [20–22]. BHRF1 protein shares 38 % primary sequence homology with human Bcl-2 and has three conserved Bcl-2 homology (BH) domains, BH1–BH3 [23]. Helices α2-α5 of BHRF1 protein form a hydrophobic surface groove which can bind BH3 domains of pro-apoptotic Bcl-2 family members such as Bim to block their pro-apoptotic ability [21, 24]. Though not expressed consistently in all EBV-associated tumors, BHRF1 transcripts can be detected in EBV-associated carcinomas [25], B cell lymphoma, T cell lymphoma [17], as well as in NK/T cell lymphoma [9]. Recent study linked BHRF1 to the transformation of B lymphocytes and lymphomagenesis [26].

In our previous study, the sequences of BARF1 and BHRF1 genes in Northern Chinese nasopharyngeal carcinoma (NPC), EBV-associated gastric carcinoma (EBVaGC) and throat washings (TWs) from healthy donors were analyzed [27, 28]. Sequences of these two genes were highly conserved. V29A strains, a BARF1 mutant subtype, showed higher prevalence in NPC, which may suggest the association between this variation and NPC [28]. As to BHRF1, there were no significantly different variations among different samples [27].

In order to clarify the sequence variation patterns of BARF1 and BHRF1 in NK/T cell lymphoma and explore whether the polymorphisms of the two early genes were associated with oncogenesis of NK/T cell lymphoma, we analyzed their sequences and compared the results with the data from our previous study and other studies involved different populations and regions.

## Results

Sixty nine samples of NK/T cell lymphoma tissues were tested by EBER 1 in situ hybridization, and 57 cases (82.6 %) were EBV positive. Because of the difficulty in tissue availability and extensive necrosis in NK/T cell lymphoma tissues, only 47 and 53 EBV-positive samples were used for detecting BARF1 and BHRF1 sequence respectively.

### Analysis of BARF1 gene sequence

**Fig. 1:**
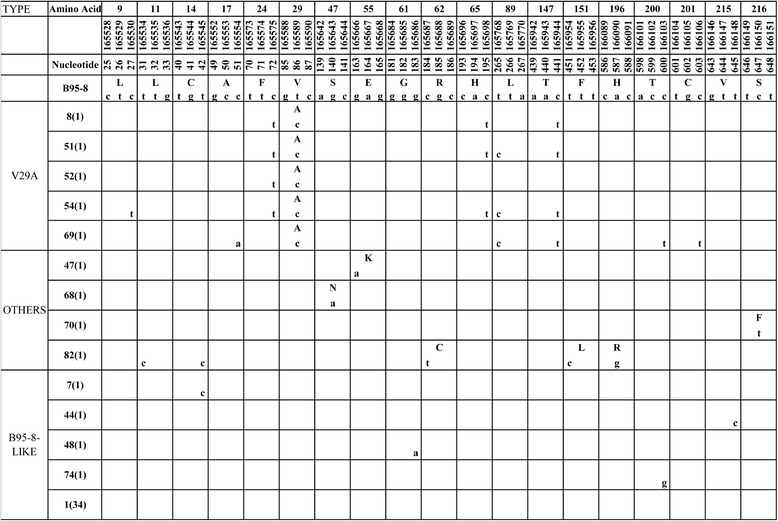
BARF1 variations in 47 NK/T cell lymphoma specimens. Numbers across the top correspond to the amino acid positions under which the B95-8 prototype amino acid and nucleotide sequence are listed. The specimens showing identical sequences to each other are listed by a representative isolate in the left column, and the numbers in the parentheses after the name denote the number of isolates carried identical sequence with the representative isolate

The sequences across the whole coding region of BARF1 gene (nt 165504–166169) were analyzed. The mutation status of BARF1 in EBV-positive NK/T cell lymphoma samples was shown in Fig. 1.

Thirty eight of 47 cases (80.9 %) had the identical amino acid sequence to B95-8 prototype. These isolates were arranged into one group and designated as B95-8 subtype.

We identified totally seven amino acid mutations and 12 silent mutations in all samples (Fig. 1). The most frequent amino acid mutation was AA 29 Val → Ala and 5 cases with this mutation were grouped into subtype V29A. 4 silent mutations (165575 nt C → T, 165698 nt C → T, 165768 nt T → C, 165944 nt C → T) were detected in more than three samples (Fig. 1).

Other amino acid or silent mutations were sporadic and most were found only in one sample. The remaining 4 isolates with no common amino acid mutations were classified as “Others” (Fig. 1).

### Distribution of BARF1 subtype in different samples

The frequency of BARF1 subtypes in NK/T cell lymphoma, NPC, EBVaGC and TWs from healthy people was summarized in Table 1. Data of NPC, EBVaGC and TWs were cited from our previous study [28]. The distribution of the BARF1 subtypes in NK/T cell lymphoma group was similar to that in EBVaGC (*χ*^2^ = 5.07, *P* > 0.05), NPC group (*χ*^2^ = 4.77, *P* > 0.05) and TWs (*χ*^2^ = 1.32, *P* > 0.05).

**Table 1:** Distribution of BARF1 and BHRF1 subtypes in NK/T, NPC, EBVaGC and TWs

Gene	Subtype	NK/T	NPC	EBVaGC	TWs
BARF1	B95-8	38 (80.9 %)	56 (70.9 %)	41 (91.1 %)	40 (87.0 %)
	V29A	5 (10.6 %)	20 (25.3 %)	0 (0 %)	2 (4.3 %)
	OTHERS	4 (8.5 %)	3 (3.8 %)	4 (8.9 %)	4 (8.7 %)
	total	47 (100 %)	79 (100 %)	45 (100 %)	46 (100 %)
BHRF1	79V88V	41 (77.4 %)	26 (66.7 %)	34 (85.0 %)	41 (77.4 %)
	79L88L	4 (7.5 %)	4 (10.2 %)	2 (5 %)	0 (0 %)
	79V88L	8 (15.1 %)	9 (23.1 %)	4 (10.0 %)	12 (22.6 %)
	total	53 (100 %)	39 (100 %)	40 (100 %)	53 (100 %)

### Sequence variations in functional domains of BARF1

**Fig. 2:**
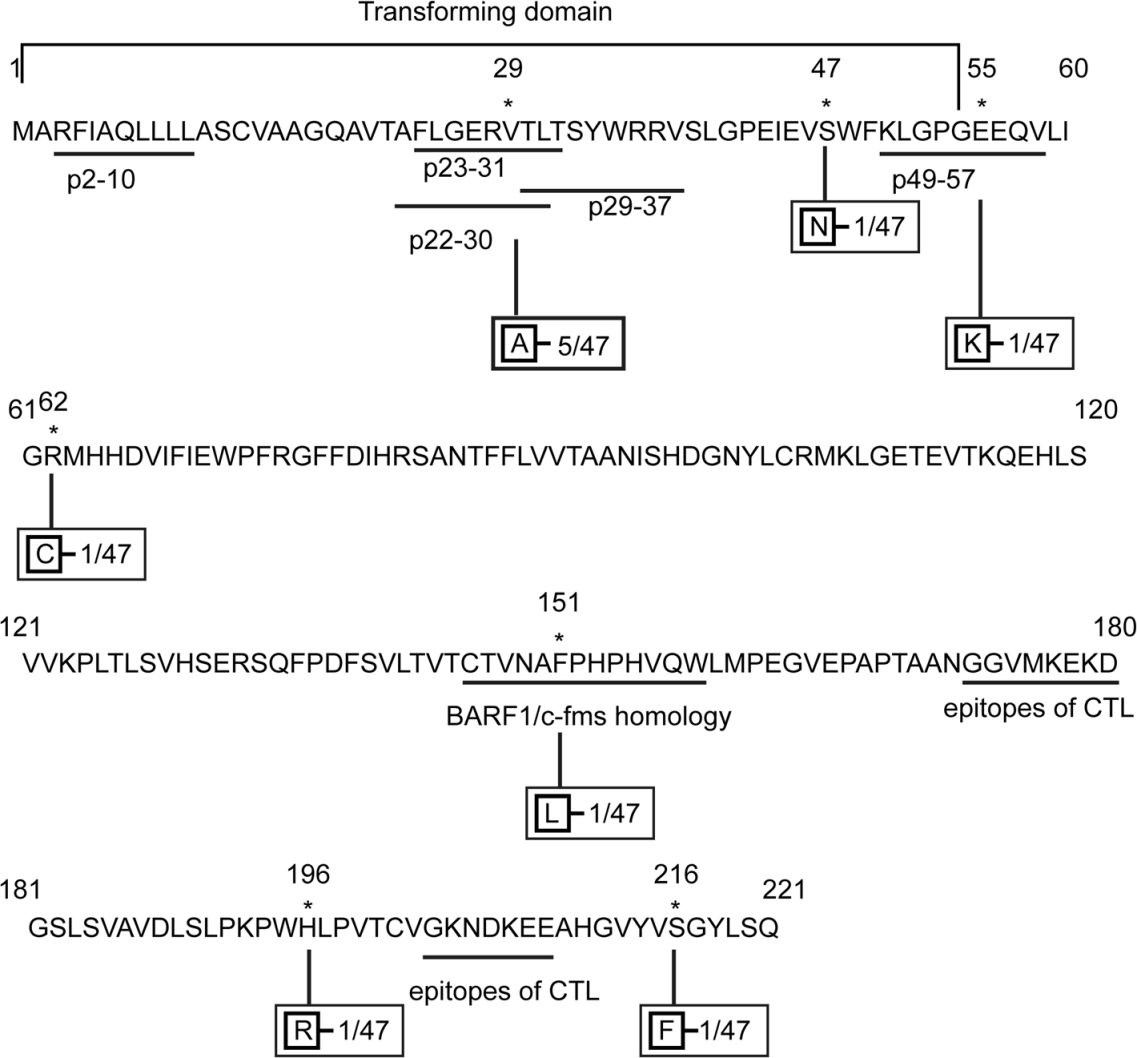
Schematic diagram of amino acid sequence variations in BARF1 protein. The amino acid sequence of BARF1 protein derived from B95-8 is listed. The previously identified BARF1/c-fms homology domain, transforming domain and CTL epitopes of BARF1 are underlined at the corresponding regions. Numbers indicate amino acid positions; asterisks indicate mutant amino acids. The mutated amino acid residues to B95-8 are indicated in boxes. Frequency of the mutations are also showed

Most amino acid mutations exhibited in the first 60 amino acids of BARF1 protein, where essential transforming domain (amino acids 1–54) [10] and 5 HLA-A*0201-restricted CTL epitopes [15] were located (Fig. 2). The hottest V29A was detected at position 6 of epitope p23–31 or position 7 of p22–30 in 5 of 47 NK/T cell lymphoma isolates. Epitopes p2–10, p29–37 and p49–57 were highly conserved except for a E → K change at position 7 of AA 49–57 in 1 isolate (Fig. 2). At the transforming domain, only 1 more amino acid mutation was obtained in 1 isolate except for the V29A change (Fig. 2). Totally, this amino terminal region was conserved in 40 of 47 (85.1 %) isolates. The homology domain of c-fms is located at BARF1 AA 146–158. BARF1 can affect the function of hCSF-1 by combining with hCSF-1 [13]. This homology domain was also shown to be conserved in all NK/T cell lymphoma samples except F151L in 1 sample (Fig. 2). No mutations of the two epitopes for CTL at AA 172–180 (NGGVMKEKD) and 203-209(GKNDKEE) have been observed in NK/T lymphoma specimens.

### Analysis of BHRF1 gene sequence

**Fig. 3:**
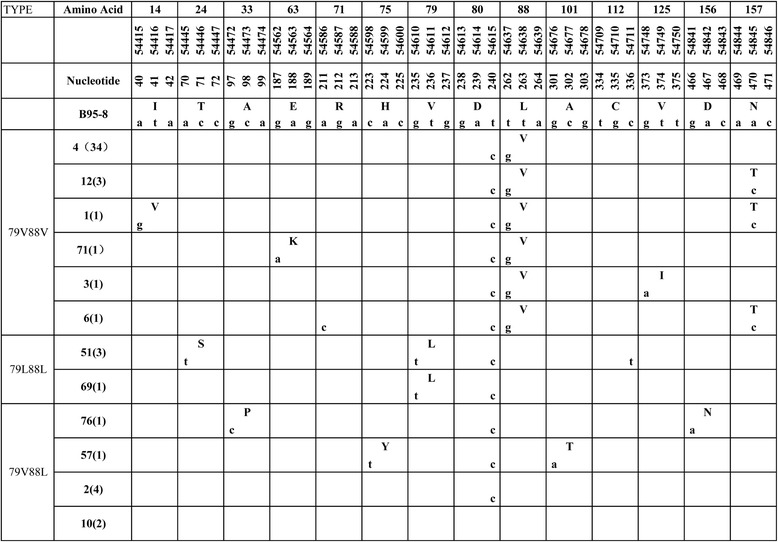
BHRF1 variations in 53 NK/T cell lymphoma specimens. Numbers across the top correspond to the amino acid positions under which the B95-8 prototype amino acid and nucleotide sequence are listed. Different patterns are noted to the left column, while the specimens showing identical sequences to each other are listed by a representative isolate in the second column. The followed numbers in the parentheses denote the amount of the identical sequences

BHRF1 gene sequence was analyzed across the whole coding region (nt 54376–54951). In contrast to BARF1, most EBV isolates exhibited amino acid mutations in BHRF1 with only 6 sample identical to the prototype B95-8. 47 of 53 cases (88.7 %) carried amino acid mutations. We identified 11 amino acid mutations and 3 silent mutations in all samples (Fig. 3). The amino acid mutation 88 Leu → Val and silent mutation 54615 nt T → C were the most frequent, carried by 41 (77.4 %, 41/53) and 51 (96.2 %, 51/53) samples. Other mutations distributed rather sporadically.

**Fig. 4:**
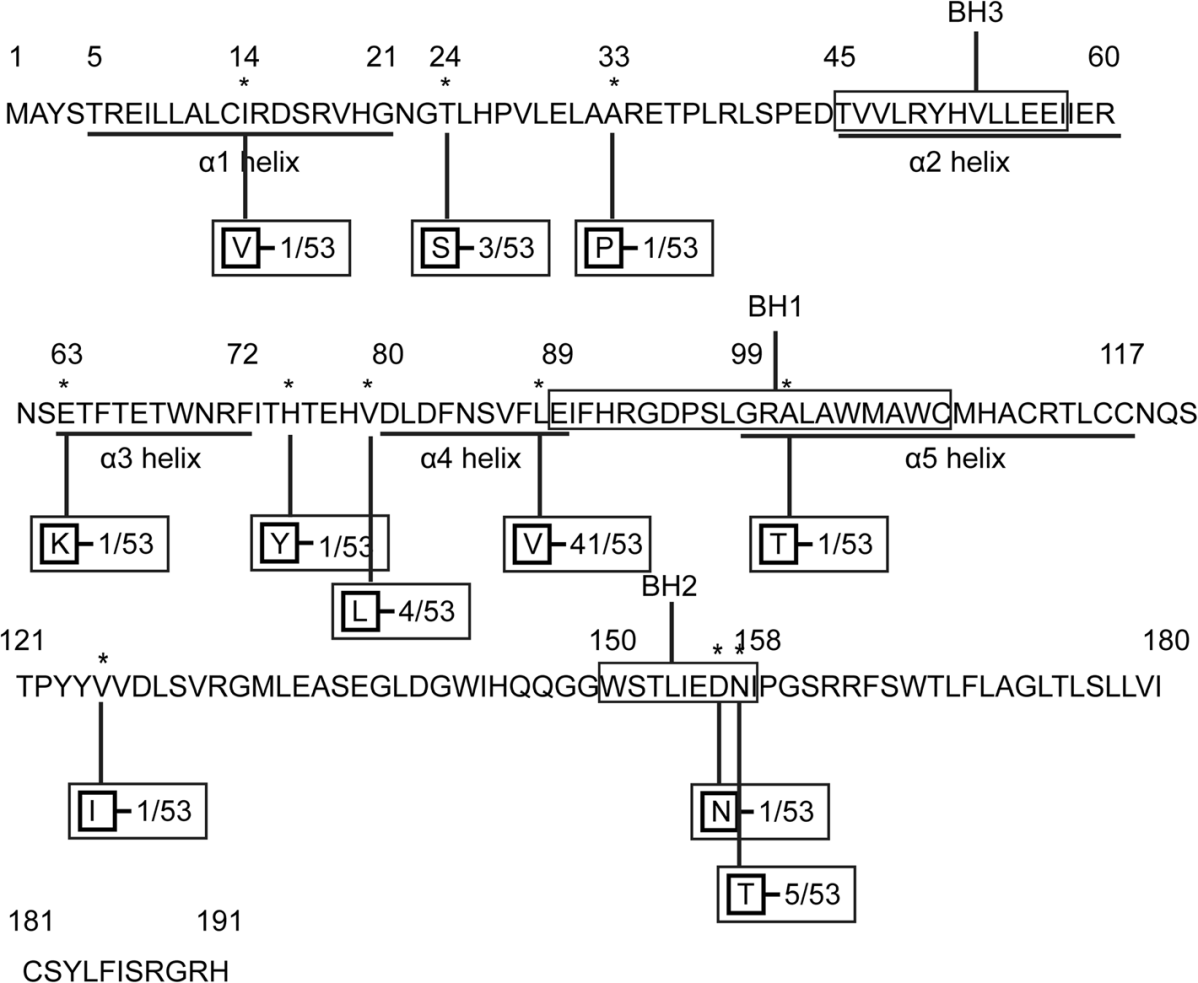
Schematic diagram of amino acid sequence variations in BHRF1 protein. The amino acid sequence of BHRF1 protein derived from B95-8 is listed. The previously identified BH1-3 domain are put into the rectangles. α2-α5 helices are underlined at the corresponding regions. Numbers indicate amino acid positions; asterisks indicate mutant amino acids. The mutated amino acid residues to B95-8 are indicated in boxes. Frequencies of the mutations were also showed

Considering the conservation of amino acid mutation and the mutual exclusion of mutations at the site of AA 79 and AA 88, the NK/T cell lymphoma cases were divided into 3 subtypes: 79V88V, 79L88L and 79V88L [27], the former two subtype carrying L88V, V79L mutations respectively, while the latter one without amino acid substitutions at these two residues.

### Distribution of BHRF1 subtype in different samples

The distribution of the BHRF1 subtypes in NK/T cell lymphoma group had no significant difference with that in NPC group (*χ*^2^ = 1.20, *P* > 0.05), EBVaGC (*χ*^2^ = 0.94, *P* > 0.05) and TWs (*χ*^2^ = 4.80 *P* > 0.05) (Table 1). Data of the latter three groups were also from another study [27].

### Sequence variations in functional domains of BHRF1

The three Bcl-2 homology domains were highly conserved. BH1 (AA 89–108) had only one mutation in 1 sample. There were two amino acid substitutions in BH2 (AA 150–158) in 6 samples. BH3 (AA 45–57) were completely conserved. Totally, BH domains were conserved in 46 of 53 (86.8 %) NK/T cell lymphoma samples. 50 samples (94.3 %) carried conservative amino acid sequence in α1 (AA 5–21), α2 (AA 45–60), α3 (AA 63–72) and α5 (AA 99–117) helix. α4 helix had the dominant mutation L88V in 41 samples (77.4 %) (Fig. 4).

## Discussion

BARF1 and BHRF1 are two important EBV genes closely related to the oncogenicity of the virus. Mutations may affect their biological activities and the pathogenicity of EBV. Only small-scale studies have reported gene variations in these two genes from different geographical area and distinct EBV-related disorders [8, 25, 29–31]. The gene polymorphisms of BARF1 and BHRF1 have been investigated in NPC and EBVaGC from Northern China [27, 28], but little is known in NK/T cell lymphoma. To our knowledge, this is the first study to explore sequences of EBV early genes BARF1 and BHRF1 in NK/T cell lymphoma.

### Comparison of BARF1 among different diseases and different regions

In the present study, the AA sequences of BARF1 in 38 of 47 (80.9 %) NK/T cell lymphoma cases were identical to the prototype B95-8, suggesting BARF1 was highly conserved. This result was similar to our previous data from NPC, EBVaGC and TWs from healthy donors in the same area [28]. Some other studies also showed that B95-8 subtype of BARF1 was predominant, involving 4 of 6 (66.7 %) cell lines established from EBV-associated NK/T cell disorders in Japan [8], 10 of 15 (66.7 %) non-NPC patients in Europe [29] and 28 of 28 (100 %) NPC patients and healthy donors from Italy [30]. All the above areas are NPC non-endemic. However, in Indonesia, a well-known NPC endemic area, it was not the case. 45 of 56 (80.3 %) NPC isolates and 2 of 5 (40.0 %) healthy lymphoblastoid cell lines (LCL) showed amino acid mutations in BARF1.

Despite the scarcity of mutations with only seven amino acid mutations exhibited in nine samples, the relatively hot mutational spot was V29A detected in 5 of 47 (10.6 %) samples, accompanied with one or more of four silent mutations (165575 nt C → T, 165698 nt C → T, 165768 nt T → C, 165944 nt C → T). We have detected V29A BARF1 subtytpe in 20 of 79 (25.3 %) NPC, 0 of 45 (0 %) EBVaGC and 2 of 46 (4.3 %) TWs from healthy donors [28]. The frequency of this subtype in NK/T cell lymphoma was similar to that in other samples in Northern China. In Indonesia, V29A mutants was the most dominant in NPC (80.3 % of all NPC), but it was statistically insignificant compared to the mutation rate of 40.0 % in LCLs from healthy EBV carriers (*p* = 0.074) [29]. These findings suggest that variations in BARF1 in tumor samples are similar to those in the background population and were geographic-associated rather than tumor-specific. However, V29A subtype in Indonesian NPC isolates showed higher frequency (78.6 %, 44/56) [29] than in NPC isolates from NPC non-endemic areas including Northern China (25.3 %, 20/79) [28], Italy (0/14) [30], and in non-NPC isolates from Europe (33.3 %, 5/15) [29] and Northern China (2.2 %, 2/91) [28]. Furthermore, the V29A subtype showed a higher prevalence in NPC (25.3 %, 20/79) than in EBVaGC (0/45) and healthy donors (4.3 %, 2/46) in Northern China. This may also suggest association of this mutated subtype with NPC. To date, little data is available about the sequence mutations of BARF1 gene in NK/T cell lymphoma, so we couldn’t obtain valuable information from other areas of NK/T cell lymphoma to compare with our results.

BARF1 were conserved in most EBV strains. This may contribute to the maintaining of its biological function. Almost all of the few amino acid mutations were outside the functional domains. Conservation of the c-fms domain contributes to the immunomodulation of BARF1 to help the virus evade immune surveillance of the host. Using the crystal structure of hexameric B95-8 derived BARF1 [32], Hutajulu et al. [29] reported that the V29A mutation in the transforming domain are not considered to change the structure of the protein, so may have little effect on its function. The conserved transforming domain may prompt malignant transformation of the infected cells. Considering the presence of highly conserved CTL epitopes, BARF1 may be used as a useful target for immunotherapy to EBV-associated tumors.

### Comparison of BHRF1 among different diseases and different regions

We found that most EBV isolates (88.7 %, 47/53) in NK/T cell lymphoma showed amino acid mutations. These mutations were highly conservative with L88V detected in 41 (41/53, 77.4 %) samples. The findings were similar to those from NPC, EBVaGC and TWs from healthy donors in the same area [28]. Two other small scale studies also detected conserved amino acid mutations in most samples [25, 31].

In Northern China, amino acid mutation L88V has been found in 26 of 39 (66.7 %) NPC, 34 (85.0 %) of 40 EBVaGC, and 41 (77.4 %) of 53 TW samples from healthy donors [28]. Together with our findings in NK/T cell lymphoma in the same area, these data suggest that 79V88V BHRF1 subtype was the dominant EBV substrain in this area and no disease-restricted mutations were found. Liu et al. [25] detected more V79L88L subtypes than 79V88V in Taiwanese [57.1 % (8/14) vs. 42.9 % (6/14)]. The difference between the two studies may be due to the relative small size of samples of the latter. Otherwise, it may reflect different dominant EBV strains in different geographic areas. In their study, 8 of 11 NPC tissues exhibited V79L88L subtypes, while all the 3 benign lymphatic disorders carried 79V88V. But it couldn’t reflect the possibility that particular mutations were associated with NPC, because of the limitation of sample size. In another study [31], BHRF1 sequences were analyzed in 15 EBV strains from different samples and different regions: 5 Chinese samples [2 normal lymphoblastoid cell lines (LCL), 2 NPC patient LCL, and 1 NPC biopsy], 6 African samples (4 BL cell lines, 1 normal LCL, and 1 SCID mouse passaged NPC), and 4 European HL samples. V79L88L and 79V88V were detected respectively in five samples (2 African BL cell lines, 1 African normal LCL, 1 Chinese normal LCL and 1 Chinese NPC biopsy) and 2 samples (2 Chinese NPC patient LCL). This study showed that BHRF1 is highly conserved between EBV isolates no matter what geographical area or EBV-related samples they came from. All the above data suggest that the conserved mutations of BHRF1 gene are not associated with particular EBV-related diseases. And whether BHRF1 mutations reflect different EBV strains popular in diverse geographical regions needs much more data to support.

In mammalian cells, Bcl-2 protein plays its anti-apoptotic role by binding with pro-apoptotic proteins such as Bim, Bax and Bak [33]. BHRF1 is isogenous with the human anti-apoptosis gene Bcl-2 in structure and function [24]. There are three conserved Bcl-2 homology (BH) domains, BH1–BH3, in BHRF1 amino acid sequence. The three-dimensional structure of BHRF1 is similar to that of Bcl-2 protein with six α helices [23]. Helix α2-α5 of BHRF1 protein form a hydrophobic groove which can bind BH3 domains of Bim to block its pro-apoptotic ability [21, 24]. BHRF1 has similar function to Bcl-2 and can protect EBV-infected cells from apoptosis [20–22]. It may facilitate the establishment of virus persistence and prompt oncogenesis.

Most amino acid mutations of BHRF1 in NK/T cell lymphoma were sporadic and outside the important domains. Most strains were conserved in all BHRF1 functional domains except α4 helix. Though α4 helix carried the dominant mutation L88V in 41 samples (77.4 %), considering the similar properties of valine and leucine: both are neutral and hydrophobic, this substitution was not expected to have any effects [25]. Khanim et al. [31] reported that despite several amino acid changes in the BHRF1 of some EBV isolates, its ability to resist cis-platin induced apoptosis was conserved. The highly conserved nature of BHRF1 among different EBV isolates at both the sequence and functional level supports the proposed important role of BHRF1 in delaying cell death to establish virus persistence and thereby contributing to tumor formation.

## Conclusions

In summary, the data from the present study indicated that the sequences of the two EBV early oncogenes BARF1 and BHRF1 were highly conserved. This is important to maintain their function and biological activity in a variety of pathophysiological conditions. The similar distributions of different subtypes among different patients and healthy donors suggest that variations of BARF1 and BHRF1 were not associated with pathogenesis of specific EBV-associated malignancies. Recent and ongoing developments in Deep Sequencing may help to reveal the relevance of genomic variation in EBV isolates and their relation to distinct EBV-driven diseases.

## Materials and methods

### Case selection

Sixty nine cases of NK/T cell lymphomas were collected from the Department of Pathology of Affiliated Hospital of Qingdao University between January 2009 and December 2012. The study was approved by the Medical Ethics Committee at the Medical College of Qingdao University and the informed consents were obtained from the study participants. All the cases were reviewed and confirmed by at least two pathologists by using WHO criteria [34]. In situ hybridization (ISH) was performed with specific digoxigenin-labeled probes complementary to EBV encoded RNA nuclear transcripts as described previously [35]. 4 μm formalin-fixed, paraffin-embedded tissue sections were used for ISH and DNA extraction.

### DNA Extraction and PCR

DNA was extracted using A QIAamp DNA FFPE Tissue Kit (QIAGEN GmbH, Hilden, Germany) from the paraffin-embedded tumor tissue sections.

Nested-polymerase chain reaction (nested-PCR) technique was performed as previously described [27, 28]. In each set of PCR, DNA from EBV-positive B95-8 cell lines and EBV-negative Ramos cell lines were used as positive and negative controls, respectively. The PCR products were analyzed by electrophoresis through 1.2 % agarose gel.

### DNA sequence analysis

Forty five micro liters products of the second round of PCR were directly sequenced in both directions with primers used in the second round PCR by means of a Prism ready reaction Dyedeoxy terminator cycle sequencing kit. After sequencing, the sequence data of the two genes were checked for homology using BLAST (National Center for Biotechnology Information, http://www.ncbi.nlm.nih.gov/ and were compared with the B95-8 prototype strain [GenBank: V01555]. Alignments between sequences were analyzed using DNA Star software (DNASTAR, Inc., version 5.0).

### Statistical analysis

The *χ*2 test was performed to analyze the difference of the EBV genotypes among the NK/T cell lymphoma and other EBV associated diseases studied previously [27, 28]. Data were considered to be statistically significant if *P*-value <0.05.

## Abbreviations

EBV: Epstein-Barr virus
BARF1: BamHI-A rightward open reading frame 1
BHRF1: BamHI-H rightward open reading frame 1
NPC: Nasopharyngeal carcinoma
EBVaGC: EBV-associated gastric carcinoma
TWs: Throat washings
ISH: In situ hybridization
nested-PCR: Nested-polymerase chain reaction
EBER1: EBV-encoded small RNA 1
LMP1: Latent membrane protein 1
EBNA1: Epstein-Barr virus nuclear antigen 1
BL: Burkitt lymphoma
HL: Hodgkin lymphoma
hCSF-1: Human colony-stimulating factor 1
CTL: Cytotoxic T lymphocyte
ADCC: Antibody-dependent cell-mediated cytotoxicity
